# Successful Treatment of Hepatitis C with Simeprevir, Sofosbuvir, and Ribavirin in an HIV Coinfected Liver Transplant Patient with Advanced Chronic Kidney Disease

**DOI:** 10.1155/2016/8372835

**Published:** 2016-03-30

**Authors:** Anna Maruyama, Trana Hussaini, Nilufar Partovi, Siegfried R. Erb, Vladimir Marquez Azalgara, Nadia Zalunardo, Neora Pick, Mark Hull, Eric M. Yoshida

**Affiliations:** ^1^Faculty of Pharmaceutical Sciences, University of British Columbia, Vancouver, BC, Canada; ^2^Division of Gastroenterology, University of British Columbia, Vancouver, BC, Canada; ^3^Division of Nephrology, University of British Columbia, Vancouver, BC, Canada; ^4^Division of AIDS, University of British Columbia, Vancouver, BC, Canada

## Abstract

Although major advances have occurred in treating patients with hepatitis C virus (HCV) with the development of new direct-acting antivirals (DAAs), treatment of liver transplant recipients with HCV, human immunodeficiency virus (HIV) coinfection, and renal disease is challenging due to the lack of efficacy and safety data in this population. We report a case of successful HCV therapy in a postliver transplant HIV coinfected patient, with stage 4 chronic kidney disease, using an all-oral regimen of simeprevir, sofosbuvir, and ribavirin. The 51-year-old male achieved SVR24, and no specific HIV-related or transplant-related adverse events were documented during the treatment period. The new DAAs show promise for HIV coinfected patients and those with severe to end-stage renal disease (ESRD); however, robust clinical trials or large cohort studies will need to be conducted to confirm the efficacy and safety of these newer agents in this setting.

## 1. Introduction

It is estimated that 25–30% of patients who have human immunodeficiency virus (HIV) infection also have hepatitis C virus (HCV) infection [1–3]. Coinfection with both viruses can accelerate the progression of liver disease leading to fibrosis, cirrhosis, decompensation, hepatocellular carcinoma, and potentially death [1–3]. HCV can also lead to extrahepatic manifestations such as renal disease, for example, membranoproliferative glomerulonephritis with or without cryoglobulinemia [4] that can eventually progress to end-stage renal disease (ESRD). Previous standard therapy of HCV infection, a combination of peginterferon and ribavirin, came with intolerable adverse effects and poor cure rates especially in the HIV coinfected population [5]. Although major advances have occurred in the treatment of HCV with the development of new direct-acting antivirals (DAAs), treatment in patients with liver transplantation and those with severe renal disease is fraught with challenges due to the lack of efficacy and safety data in these specific patient populations. Here, we report, to our knowledge, the first case of successful HCV therapy after liver transplantation in an HIV coinfected patient with stage 4 chronic kidney disease (GFR 15–29 mL/min) using an all-oral regimen of simeprevir, sofosbuvir, and ribavirin for 24 weeks.

## 2. Case Report

The patient was a 51-year-old HIV positive male who received deceased liver transplantation for end-stage liver disease secondary to HCV genotype 1a. In 1998, 2000, and 2005 prior to his transplant, his HCV infection was treated with a combination of interferon or peginterferon and ribavirin. However, on all three occasions, he failed treatment because of truncated treatment duration due to significant adverse reactions, notably treatment-related cytopenias. One year after transplant, he developed moderate graft fibrosis secondary to HCV recurrence confirmed by a liver biopsy. It was decided to defer therapy at that time as treating his HCV with the available agents, the first-generation protease inhibitors telaprevir, or boceprevir in combination with peginterferon and ribavirin, was deemed potentially futile due to previous significant adverse reactions to pegylated interferon. Over the ensuing 22 months, his liver function deteriorated as his graft fibrosis progressed. Transient elastography (Fibroscan, Echosens/KNS Canada, Toronto, ON) showed advanced fibrosis with a stiffness score of 12.4 kPa (F3 to F4 fibrosis where F4 is cirrhosis). In addition, his renal function declined rapidly reaching stage 4 chronic kidney disease which prompted a renal biopsy confirming immune complex mediated membranoproliferative glomerulonephritis presumed to be secondary to HCV infection. Initiation of HCV therapy was felt to be clinically urgent to prevent progression of both hepatic and renal disease. At this time, the second-generation HCV NS3/4A protease inhibitor simeprevir and the NS5B polymerase inhibitor sofosbuvir had recently been approved by Health Canada. The lack of safety data and inexperience using simeprevir and sofosbuvir in the setting of liver transplantation, HIV coinfection, and near-end-stage renal disease made treating this patient very challenging. A multidisciplinary team was involved in deciding to treat this patient, and informed consent was obtained from the patient. Both simeprevir and sofosbuvir were obtained through their respective manufacturers on compassionate grounds.

His antiviral and immunosuppressant medications consisted of 400 mg raltegrevir, 600 mg abacvir, 300 mg lamivudine, and 0.5 mg tacrolimus once daily and 500 mg mycophenolate twice daily. The patient also had a number of other comorbidities (epilepsy, hypertension, hypothyroidism, and type 2 diabetes) that were treated with the appropriate medications. His liver biochemistry prior to initiating HCV treatment is listed in Table 1. Additional molecular analysis of his genotype 1 HCV revealed presence of the Q80K polymorphism. At the outset of treatment, his HCV viral load was at 3,771, 626 IU/mL (lower limit of detection 15 IU/ML). His HCV treatment consisted of a 24-week regimen of simeprevir 150 mg once daily, sofosbuvir 400 mg once daily, and renally adjusted ribavirin 200 mg once daily. He was monitored as an outpatient on a weekly basis for the first month and then biweekly thereafter. The ribavirin dose was decreased to every other day at week 14 of treatment due to anemia, with hemoglobin of 86 g/L despite weekly darbepoetin administration. Ribavirin was eventually discontinued at week 15 due to persistent symptomatic anemia. Discontinuing the ribavirin did not make any difference in his hemoglobin and he continued to be maintained on weekly darbepoetin administration. His HCV viral load was undetectable by week 4 and remained undetectable 6 and 8 months following his last dose of treatment. Adverse effects consisted of nausea, insomnia, and fatigue, which were tolerated during the treatment.

Unfortunately, the patient's renal function steadily deteriorated over the course of treatment. Despite successful HCV eradication, his renal dysfunction progressed to ESRD requiring dialysis 3 months following HCV treatment completion. The nephrology service did not feel that his progression to ESRD was aggravated by the antiviral agents used to treat his HCV. From a liver perspective, there was significant improvement in his liver biochemistry, and he was deemed cured of HCV, as his viral load remained undetectable 8 months after the end of treatment.

## 3. Discussion

At the time of initiating this patient's HCV treatment, there was very little experience using the combination of simeprevir, sofosbuvir, and ribavirin in HIV coinfected patients, with postliver transplant, with severe renal impairment. The available data for HIV coinfection were for simeprevir with peginterferon and ribavirin [6], or sofosbuvir and ribavirin [7]. No data were available on either simeprevir or sofosbuvir in a postliver transplant setting. Evidence for simeprevir and sofosbuvir with or without ribavirin in treatment naïve or experienced patients with advanced fibrosis (F3 to F4) showed that the combination was well tolerated with SVR 12 or 24 rates of >93% [8]. Safety and efficacy for simeprevir and sofosbuvir had not been established in patients with creatinine clearance of less than 30 mL/min or ESRD including patients requiring dialysis [9, 10]. Ribavirin is mainly eliminated through the kidneys, and therefore dosing depends on renal function as measured by creatinine clearance. It is suggested that patients with creatinine clearance of <30 mL/min be started on a dose of 200 mg per day [11, 12]. Despite the patient's renal function being <30 mL/min, the decision to use this combination at full doses of simeprevir and sofosbuvir in addition to ribavirin 200 mg daily (dosed according to his renal function) was made as the patient and his treating team wanted to maximize the chances of cure. Full-dose simeprevir was likely acceptable since renal clearance plays an insignificant role (<1%) in the elimination of simeprevir [9]. Sofosbuvir is a prodrug that undergoes extensive intracellular metabolism to form the pharmacologically active metabolite GS-461203 via phosphorylation [10]. The active metabolite is then dephosphorylated into the inactive metabolite GS-331007 which is the predominant circulating metabolite following administration [10]. The majority of sofosbuvir is eliminated by the kidney (~81%), where 78% of this is recovered as GS-331007 and 3.5% is sofosbuvir [10]. A single dose of sofosbuvir 400 mg was studied in non-HCV infected patients with varying degrees of renal function including those with ESRD requiring dialysis [10]. Compared to subjects with normal renal function (eGFR > 80 mL/min), AUC∞ for sofosbuvir and GS-331007 were 171% and 451% higher in patients with severe renal impairment [11]. It was unknown what effect full-dose sofosbuvir would have knowing the potential for higher exposure to sofosbuvir and GS-331007. Ribavirin was challenging to dose since the patient already had anemia and poor renal function prior to initiating HCV treatment. In a trial that is still underway (*N* = 17), simeprevir 150 mg once daily combined with sofosbuvir 400 mg once daily for 12 weeks appears to be well tolerated in genotype 1-infected patients with ESRD on HD or GFR < 30 mL/min [13]. Baseline characteristics of included patients revealed that 82% were treatment naïve, 47% were cirrhotic, and 88% required dialysis. Although the results are preliminary with several patients still completing treatment or awaiting SVR12, the cure rate was 100% (*N* = 11) in patients that completed treatment and reached SVR12. Therefore, the suboptimal dosing of ribavirin which was subsequently stopped early in this patient probably did not have much of an impact on his SVR.

The patient's HCV was Q80K positive but clinical evidence available at that time revealed minimal impact on SVR when simeprevir was combined with sofosbuvir with or without ribavirin [8]. The decision to extend the duration of treatment from 12 to 24 weeks was based on the fact that this patient had HCV recurrence postliver transplant, and he was treatment experienced with advanced fibrosis. The combination of simeprevir, sofosbuvir, and ribavirin has not been studied in HIV coinfection; however, given data for each DAA in this population, similar outcomes to monoinfected patients were anticipated, and the patient would not experience any significant drug interactions based on his medication list. The team was prepared to monitor tacrolimus levels (as simeprevir can potentially decrease tacrolimus plasma concentrations up to 24%) [9]. However, because his kidney function declined during his HCV treatment, the patient's tacrolimus was discontinued and his mycophenolate was increased to 750 mg twice daily.

## 4. Conclusion

Here, we report the first successful HCV treatment in a HCV, HIV coinfected patient who was HCV treatment experienced and had advanced fibrosis and stage 4 chronic kidney disease. Full-dose simeprevir and sofosbuvir were relatively well tolerated despite significant reduction in renal function. Ribavirin was dosage adjusted and discontinued three and a half months into treatment. Although his membranoproliferative glomerulonephritis was thought to be an extrahepatic manifestation of HCV, successfully treating his HCV did not ameliorate the progression of renal disease to ESRD and dialysis. With current even newer DAAs on the forefront, more clinical trials are needed in hard-to-treat populations, including the posttransplant setting, and patients with ESRD and on hemodialysis. From a HCV-HIV coinfection perspective, although previous studies had reported that this group had worse survival posttransplant outcomes compared to HIV transplant recipients without HCV [14], our experience clearly demonstrates that this may no longer be true.

## Figures and Tables

**Table 1:** Liver biochemistry prior to initiating HCV treatment.

Albumin	19 g/L (35–50 g/L)
AST	40 U/L (0–35 U/L)
ALT	19 U/L (3–36 U/L)
ALP	165 U/L (35–100 U/L)
Bilirubin (total)	5 *μ*mol/L (<26 *μ*mol/L)
GGT	77 U/L (8–61 U/L)
Hemoglobin	83 g/L (140–174 g/L)
INR	1.0 (0.9–1.2)
Serum creatinine	257 *μ*mol/L (70–120 *μ*mol/L)
WBC	7.0 × 10^9^/L (4–10 × 10^9^/L)

AST: aspartate aminotransferase; ALT: alanine aminotransferase; ALP: alkaline phosphatase; GGT: gamma glutamyl transferase; INR: international normalized ratio; WBC: white blood cells.
